# Structure-functional changes in eNAMPT at high concentrations mediate mouse and human beta cell dysfunction in type 2 diabetes

**DOI:** 10.1007/s00125-019-05029-y

**Published:** 2019-11-15

**Authors:** Sophie R. Sayers, Rebecca L. Beavil, Nicholas H. F. Fine, Guo C. Huang, Pratik Choudhary, Kamila J. Pacholarz, Perdita E. Barran, Sam Butterworth, Charlotte E. Mills, J. Kennedy Cruickshank, Marta P. Silvestre, Sally D. Poppitt, Anne-Thea McGill, Gareth G. Lavery, David J. Hodson, Paul W. Caton

**Affiliations:** 1 Diabetes Research Group, Department of Diabetes, School of Life Course Sciences, King’s College London, Hodgkin Building, Guy’s Campus, London, SE1 1UL UK; 2 Protein Production Facility, Randall Centre for Cell and Molecular Biophysics, King’s College London, London, UK; 3 Institute of Metabolism and Systems Research (IMSR), University of Birmingham, Birmingham, UK; 4 Centre of Membrane Proteins and Receptors (COMPARE), University of Birmingham, Birmingham, UK; 5 Centre for Endocrinology, Diabetes and Metabolism, Birmingham Health Partners, Birmingham, UK; 6 Michael Barber Centre for Collaborative Mass Spectrometry, School of Chemistry, Manchester Institute of Biotechnology, Manchester, UK; 7 Division of Pharmacy and Optometry, Faculty of Biology, Medicine and Health, University of Manchester, Manchester, UK; 8 Department of Nutritional Sciences, School of Life Course Sciences, King’s College London, London, UK; 9 Nutrition Research Group, University of Reading, Reading, UK; 10 Human Nutrition Unit, School of Biological Sciences, University of Auckland, Auckland, New Zealand; 11 School of Health & Human Sciences, Southern Cross University, Lismore, NSW Australia

**Keywords:** Beta cell, eNAMPT, Extracellularnicotinamide phosphoribosyltransferase, Inflammation, Insulin secretion, NAD, Type 2 diabetes

## Abstract

**Aims/hypothesis:**

Progressive decline in functional beta cell mass is central to the development of type 2 diabetes. Elevated serum levels of extracellular nicotinamide phosphoribosyltransferase (eNAMPT) are associated with beta cell failure in type 2 diabetes and eNAMPT immuno-neutralisation improves glucose tolerance in mouse models of diabetes. Despite this, the effects of eNAMPT on functional beta cell mass are poorly elucidated, with some studies having separately reported beta cell-protective effects of eNAMPT. eNAMPT exists in structurally and functionally distinct monomeric and dimeric forms. Dimerisation is essential for the NAD-biosynthetic capacity of NAMPT. Monomeric eNAMPT does not possess NAD-biosynthetic capacity and may exert distinct NAD-independent effects. This study aimed to fully characterise the structure-functional effects of eNAMPT on pancreatic beta cell functional mass and to relate these to beta cell failure in type 2 diabetes.

**Methods:**

CD-1 mice and serum from obese humans who were without diabetes, with impaired fasting glucose (IFG) or with type 2 diabetes (from the Body Fat, Surgery and Hormone [BodyFatS&H] study) or with or at risk of developing type 2 diabetes (from the VaSera trial) were used in this study. We generated recombinant wild-type and monomeric eNAMPT to explore the effects of eNAMPT on functional beta cell mass in isolated mouse and human islets. Beta cell function was determined by static and dynamic insulin secretion and intracellular calcium microfluorimetry. NAD-biosynthetic capacity of eNAMPT was assessed by colorimetric and fluorescent assays and by native mass spectrometry. Islet cell number was determined by immunohistochemical staining for insulin, glucagon and somatostatin, with islet apoptosis determined by caspase 3/7 activity. Markers of inflammation and beta cell identity were determined by quantitative reverse transcription PCR. Total, monomeric and dimeric eNAMPT and nicotinamide mononucleotide (NMN) were evaluated by ELISA, western blot and fluorometric assay using serum from non-diabetic, glucose intolerant and type 2 diabetic individuals.

**Results:**

eNAMPT exerts bimodal and concentration- and structure-functional-dependent effects on beta cell functional mass. At low physiological concentrations (~1 ng/ml), as seen in serum from humans without diabetes, eNAMPT enhances beta cell function through NAD-dependent mechanisms, consistent with eNAMPT being present as a dimer. However, as eNAMPT concentrations rise to ~5 ng/ml, as in type 2 diabetes, eNAMPT begins to adopt a monomeric form and mediates beta cell dysfunction, reduced beta cell identity and number, increased alpha cell number and increased apoptosis, through NAD-independent proinflammatory mechanisms.

**Conclusions/interpretation:**

We have characterised a novel mechanism of beta cell dysfunction in type 2 diabetes. At low physiological levels, eNAMPT exists in dimer form and maintains beta cell function and identity through NAD-dependent mechanisms. However, as eNAMPT levels rise, as in type 2 diabetes, structure-functional changes occur resulting in marked elevation of monomeric eNAMPT, which induces a diabetic phenotype in pancreatic islets. Strategies to selectively target monomeric eNAMPT could represent promising therapeutic strategies for the treatment of type 2 diabetes.

**Electronic supplementary material:**

The online version of this article (10.1007/s00125-019-05029-y) contains peer-reviewed but unedited supplementary material, which is available to authorised users.

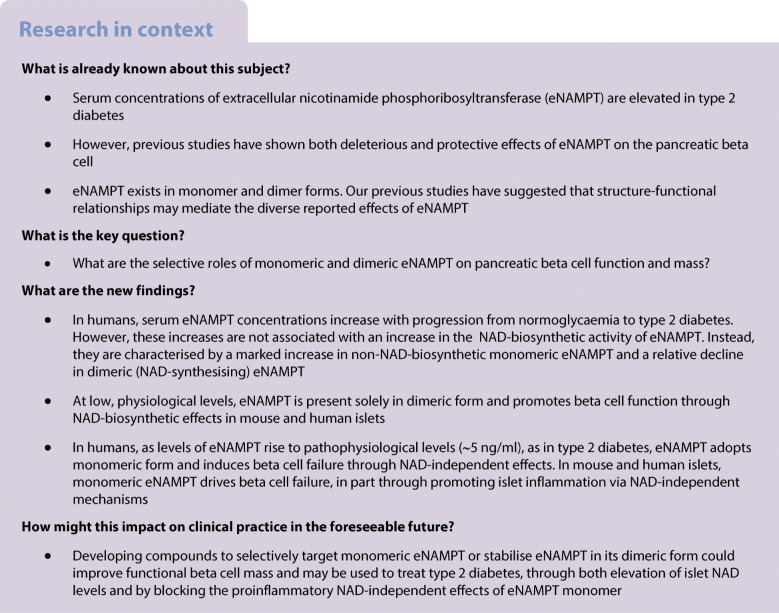



## Introduction

Elucidating the underlying mechanisms responsible for progressive decline in functional beta cell mass is essential for the design of novel treatments for type 2 diabetes [1]. The protein nicotinamide phosphoribosyltransferase (NAMPT) exists in intracellular (iNAMPT) and extracellular (eNAMPT) forms. eNAMPT (also known as visfatin/pre-B cell enhancing factor [PBEF]) is a circulating protein with several reported functions [2–4]. Similar to iNAMPT, eNAMPT may exert NAD-biosynthetic effects, whereby eNAMPT catalyses the conversion of nicotinamide to nicotinamide mononucleotide (NMN) in serum [5, 6]. NMN may subsequently be transported into the cell (directly or via conversion into nicotinamide riboside), where it is converted into NAD by nicotinamide mononucleotide adenylyltransferases (NMNATs) 1–3. However, this mechanism has been disputed and NAD-independent functions, including eNAMPT-mediated proinflammatory effects, are also reported [4, 7–9].

Serum eNAMPT levels are elevated in type 2 diabetes, in association with declining beta cell function. Our in vivo studies indicate that eNAMPT may mediate type 2 diabetes pathophysiology [10–12]. Contrasting studies report that eNAMPT and its reaction product NMN exert beta cell-protective effects by boosting cellular NAD levels [6, 13–19].

A key, but frequently overlooked factor governing eNAMPT function is the presence of structurally and functionally distinct monomeric (~52 KDa) and dimeric (~104 KDa) eNAMPT. Dimerisation is essential for NAD-biosynthetic capacity of eNAMPT [6, 20] and the eNAMPT dimer predominates in normal physiology [12, 15]. The eNAMPT monomer does not possess NAD-biosynthetic capacity and may mediate NAD-independent proinflammatory effects. Our studies suggested a specific pathophysiological role for monomeric eNAMPT in mouse models of diabetes [12]. We hypothesised that, in type 2 diabetes, eNAMPT monomer is selectively elevated, which exerts NAD-independent proinflammatory effects, and that this, combined with the loss of eNAMPT’s NAD-biosynthetic capacity, plays a key role in disease pathophysiology.

However, whether eNAMPT monomer is elevated in type 2 diabetes and to what extent the structurally distinct forms of eNAMPT directly affect the pancreatic beta cell are unknown. Previous studies, including our own, have examined NAD-boosting effects of NMN on beta cell function [6, 13, 14], or have examined supraphysiological concentrations or acute effects of eNAMPT [21, 22], neither of which accurately mimic in vivo (patho)physiology. Of perhaps greater importance, the selective effects of monomeric and dimeric eNAMPT on beta cell health have not been examined.

We used isolated mouse and human islets, and serum from humans with type 2 diabetes, to specifically characterise the effects of monomeric and dimeric eNAMPT on pancreatic beta cell functional mass.

## Methods

### Animals

Eight-week-old male CD-1 mice (28–33 g; Envigo, Blackthorn, UK) were housed in 12 h light/dark cycle, temperature-controlled conditions with ad libitum access to standard mouse chow (Envigo) and water. Procedures were performed in accordance with UK Home Office regulations (Animal Scientific Procedures Act, 1986).

### Human samples

Human serum was obtained from obese individuals without diabetes, (BMI >30 kg/m^2^; fasting plasma glucose [FPG] <5.6 mmol/l; *n* = 13 individuals), with impaired fasting glucose (IFG; BMI >30 kg/m^2^; FPG 5.6–6.9 mmol/l; *n* = 15 individuals)) or with type 2 diabetes (BMI >30 kg/m^2^; FPG >7.0 mmol/l; *n* = 27 individuals), as part of the Body Fat, Surgery and Hormone (BodyFatS&H) study (University of Auckland, Auckland, New Zealand) and from obese individuals with or at risk of developing type 2 diabetes as part of the VaSera trial (St Thomas’ Hospital, London) [23, 24]. Study participants gave informed consent. Investigations were approved by the Northern Regional Ethics Committee (NREC), Auckland, New Zealand (BodyFatS&H) and the National Research Ethics Service (NRES) Committee, London Central (VaSera). See electronic supplementary material (ESM) Methods for further details.

### Native mass spectrometry

Native mass spectrometry was conducted using the Synapt G2S high definition mass spectrometer (HDMS; Waters, Manchester, UK) (see ESM Methods).

### eNAMPT protein generation

Recombinant wild-type (WT) eNAMPT (eNAMPT-WT) and mutant SS^199^/^200^DD (eNAMPT monomer) were produced in *Escherichia coli*. See ESM Methods for details.

### Pancreatic islet isolation

Mouse islets were isolated between 9:00 and 10:00 hours [25]. Human islets were isolated from heart-beating non-diabetic donors, with ethical approval, at the King’s College Hospital Human Islet Isolation Unit (London, UK) [25] (see ESM Human Islets Checklist for further details). All isolated islets were incubated overnight (37°C, 5% CO_2_) prior to treatments (ESM Methods). RPMI media was used for all incubations. Islets were size-matched prior to treatments. Mouse and human islets were treated with eNAMPT (WT or monomer; 0.1–5 ng/ml) or NMN (100 μmol/l) for 24, 48 or 72 h. For inflammatory pathway inhibition, mouse islets were incubated with 1 ng/ml eNAMPT monomer or 5 ng/ml eNAMPT-WT, in combination with inhibitors of signal transducer and activator of transcription 3 (STAT3; using NCS74859; 50 μmol/l), P38-mitogen-activated protein kinase (MAPK; using SB203580; 1 μmol/l), NF-κB (using BAY 11-7082; 1 μmol/l) or c-Jun N-terminal kinase (JNK; using SP600125; 50 μmol/l), (Tocris Biosciences, Abingdon, UK). For CD73 inhibition, mouse islets were incubated with 0.5–5 ng/ml eNAMPT-WT for 48 h in combination with adenosine 5′-(α,β-methylene)diphosphate (AMP-CP; 1 μmol/l; Sigma-Aldrich, Poole, UK)

### Static and dynamic glucose-stimulated insulin secretion

Static or dynamic insulin secretion was assessed in response to 2 mmol/l or 20 mmol/l glucose exposure, as previously described [25]. Secreted insulin was measured by in-house ^125^I radioimmunoassay [26] (see ESM Methods).

### Endotoxin measurement

Endotoxin concentrations were measured in recombinant eNAMPT preparations using the Pierce LAL Chromogenic Endotoxin Quantitation Kit (ThermoFisher Scientific, Altrincham, UK).

### In vitro islet immunostaining

Mouse islets were pelleted and fixed in 4% (vol./vol.) buffered formalin. Sections (5 μmol/l) were incubated with anti-guinea pig insulin (Dako, Stockport, UK; catalogue no. A0564), anti-rabbit glucagon (Abcam, Cambridge, UK; ab92517) or anti-rat somatostatin (Abcam; ab30788) (1:100 for all; K) and DAPI (ThermoFisher). Secondary antibodies used were AlexaFluor 488-labelled donkey anti-rat (712-545-1250), AlexaFluor 594-labelled donkey anti-guinea pig (06-585-148) and AlexaFluor 488-labelled donkey anti-rabbit (711-545-152) (1:200 for all; Jackson ImmunoResearch, Cambridge UK). Sections were mounted on glass slides and captured on a Nikon TE2000 fluorescent microscope (Nikon Instruments, Melville, NY, USA) and quantified using ImageJ (https://imagej.nih.gov/ij/download.html; accessed December 2017–June 2018).

### Islet apoptosis

Mouse islets were treated with eNAMPT with or without cytokine cocktail (0.05 U/μl IL-1β, 1 U/μl TNF-α and 1 U/μl IFNγ; 48 h). Apoptosis was determined by the Caspase-Glo 3/7 luminescent assay (Promega, Southampton, UK).

### Quantitative reverse transcription PCR

Gene expression was determined by SYBR Green quantitative reverse transcription PCR using ΔΔC_t_ methodology and normalised against glyceraldehyde 3-phosphate dehydrogenase (GAPDH) (QuantiTect; Qiagen, Manchester, UK). See ESM Methods and ESM Table 1.

### Intracellular calcium

Whole mouse islets, incubated in HEPES bicarbonate buffer (containing 2–20 mmol/l glucose), were loaded with Fura-2 AM and imaged using a Nikon Ti-E microscope equipped with FuraLEDs (Cairn Research, Faversham, UK) (excitation, 340/385 nm; emission, 470–550 nm).

### MIN6 cell culture

MIN6 cells (mycoplasma free; obtained from J. I. Miyazaki, Osaka University, Osaka, Japan [27, 28]) were cultured at 37°C in DMEM (25 mmol/l glucose; 2 mmol/l glutamine, 10% FBS (vol./vol.), 100 U/ml penicillin, 100 μg/ml streptomycin) and treated with eNAMPT (1–5 ng/ml WT; 1 ng/ml monomer) or NMN (100 μmol/l) (48 h; Sigma-Aldrich), prior to NAD and NMN measurements.

### eNAMPT, NAD and NMN measurements

Total serum eNAMPT was measured by ELISA (Adipogen, Seoul, South Korea). MIN6 intracellular NAD and NMN levels were measured by an NAD/NADH Quantification Kit, (Sigma-Aldrich) and an in-house fluorometric assay, respectively [29, 30] (see ESM Methods).

### eNAMPT Immunoblotting

Serum eNAMPT monomer and dimer were measured by immunoblotting, under non-reducing conditions, as previously described [31]. To ensure equivalent loading of each lane, serum protein was normalised to 10 μg and an equal volume (30 μl) was added to each well (see ESM Methods).

### Data analysis

Data are expressed as mean ± SEM. Significance was tested using one- or two-way ANOVA with Tukey’s or Sidak’s post hoc test, using GraphPad PRISM 7 software (San Diego, CA, USA). Experimenters were not blind to group assignment and outcome assessment.

## Results

### Serum eNAMPT monomer concentrations are elevated in type 2 diabetes and are positively correlated with increased HbA_1c_

**Fig. 1:**
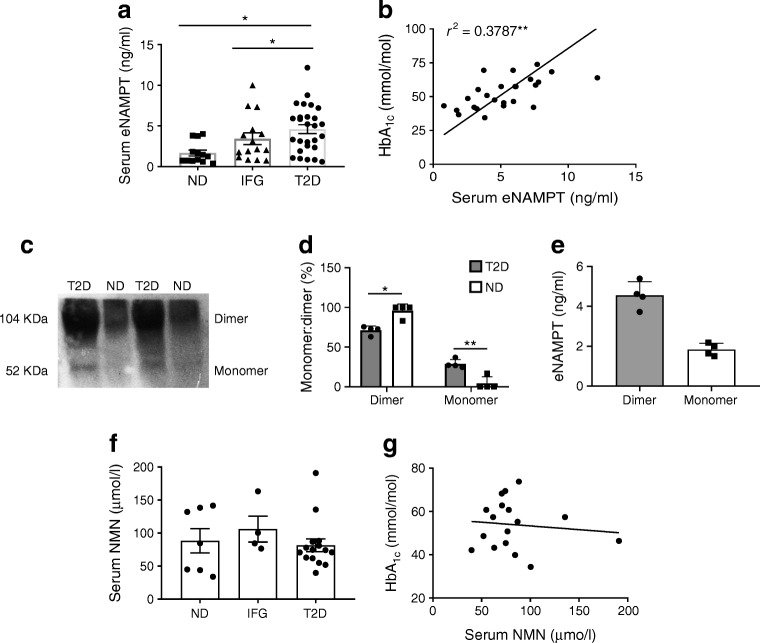
Serum eNAMPT levels are increased in type 2 diabetes and are associated with increased HbA_1c_. Serum was collected from obese individuals without diabetes (non-diabetic [ND]), with IFG and with type 2 diabetes (T2D), and eNAMPT was measured by ELISA or immunoblot. (**a**) Serum eNAMPT (data generated from BodyFatS&H and VaSera trial samples); (**b**) Serum eNAMPT vs HbA_1c_ (data generated from VaSera trial only). (**c**–**e**) Western blot of serum eNAMPT monomer and dimer (**c**) and quantification of percentage ratio of eNAMPT monomer and dimer in serum from non-diabetic and type 2 diabetic individuals (**d**) and concentration of eNAMPT monomer and dimer in serum from individuals with type 2 diabetes only (**e**). In (**c**–**e**): *n* = 4; data generated from BodyFatS&H and VaSera trial samples. (**f**) Serum NMN and (**g**) serum NMN vs HbA_1c_ (data generated from VaSera trial only). *n* values differ for NMN measurements due to limited availability of some samples. Data are expressed as means ± SEM. *p < 0.05, **p < 0.01, by one-way ANOVA with Tukey’s post hoc test, or Pearson correlation

We first demonstrated that serum eNAMPT concentrations increased with progression of type 2 diabetes (Fig. 1a): in obese non-diabetic individuals, serum eNAMPT concentrations were 1.7 ± 0.4 ng/ml, rising to 3.4 ± 0.7 ng/ml in individuals with IFG and 4.6 ± 0.6 ng/ml in type 2 diabetes (*p* < 0.05 vs non-diabetic for both IFG and type 2 diabetes). These concentrations are similar to those observed in previous studies [32–34]. Increasing eNAMPT concentrations strongly correlated (*r*^2^ = 0.3787; *p* < 0.01) with increasing HbA_1c_ (mmol/mol), indicating a role of eNAMPT in mediating poor glycaemic control (Fig. 1b). Next, we specifically measured serum eNAMPT monomer and dimer by immunoblotting under non-reducing conditions. eNAMPT monomer was almost absent in serum from individuals without diabetes, but increased markedly in type 2 diabetes. Specifically, in individuals without diabetes, serum eNAMPT was 95.8% dimer and 4.2% monomer. However, in type 2 diabetes, serum eNAMPT was 71% dimer and 29% monomer, which translated into a mean concentration of 1.83 ng/ml eNAMPT monomer (Fig. 1c–e). Increasing eNAMPT concentrations did not correlate with serum NMN levels and NMN was not associated with HbA_1c_ (Fig. 1f, g). This suggests a selective pathophysiological role for eNAMPT monomer in type 2 diabetes. Neither eNAMPT nor NMN correlated with BMI, age or serum insulin (ESM Fig. 1).

### eNAMPT exerts bimodal effects on beta cell function in mouse and human pancreatic islets

Increased HbA_1c_ is indicative of poor glycaemic control and deteriorating beta cell function. To assess the (patho)physiological effects of eNAMPT on beta cells, we used serum eNAMPT concentrations (as detected in Fig. 1a and [12]) and exposed isolated islets to physiological (1 ng/ml) and pathophysiological (5 ng/ml) eNAMPT. To account for batch-to-batch differences between commercially available eNAMPT [8], we generated recombinant wild-type eNAMPT (eNAMPT-WT). To specifically examine eNAMPT monomer, we mutated Ser^199/200^ (key residues in the NAMPT binding interface [6, 20]) to aspartic acid. This generated a 52 KDa protein (eNAMPT monomer), which neither dimerised correctly, as demonstrated by size-exclusion chromatography and native MS, nor synthesised cellular NMN and NAD (ESM Fig. 2a–e). Neither eNAMPT-WT nor eNAMPT monomer contained notable endotoxin concentrations (ESM Fig. 2f)

**Fig. 2:**
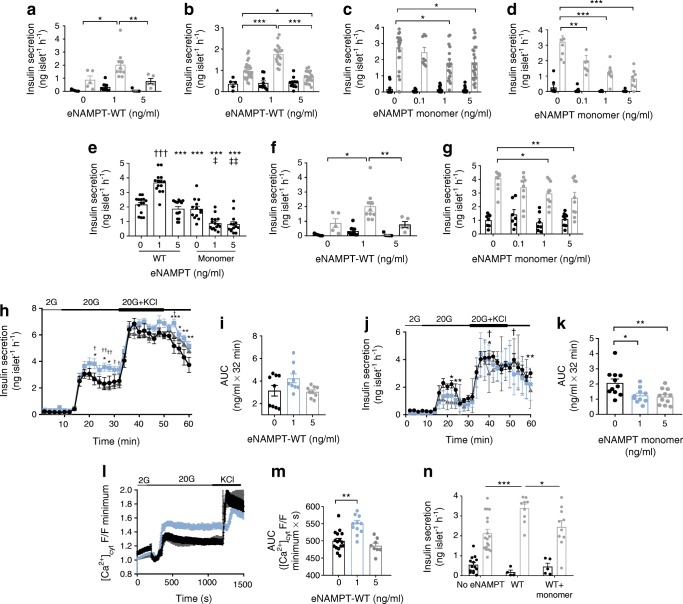
GSIS has a bimodal response to eNAMPT. (**a**–**d**) Static insulin secretion in response to basal (2 mmol/l) or 20 mmol/l glucose was assessed in isolated mouse islets incubated with 1 or 5 ng/ml eNAMPT-WT for (**a**) 24 h and (**b**) 48 h, or with 0.1, 1 or 5 ng/ml eNAMPT monomer for (**c**) 24 h and (**d**) 48 h. (**e**) Static insulin secretion in response to 20 mmol/l glucose was assessed in isolated mouse islets incubated with 1 or 5 ng/ml eNAMPT-WT and eNAMPT monomer. (**f**, **g**) Static insulin secretion in response to basal (2 mmol/l) or 20 mmol/l glucose was assessed in isolated human islets incubated with (**f**) 1 or 5 ng/ml eNAMPT-WT or (**g**) 0.1, 1 or 5 ng/ml eNAMPT monomer for 48 h. For (**a**–**g**): black bars, 2 mmol/l glucose; grey bars, 20 mmol/l glucose. *n* = 5–28, where an *n* of 1 equals five islets per incubation tube, repeated 8–10 times. (**h**–**k**) Dynamic insulin secretion was assessed in isolated mouse islets incubated with (**h**, **i**) 1 or 5 ng/ml eNAMPT-WT or (**j**, **k**) with 1 or 5 ng/ml eNAMPT monomer for 48 h by perifusion with 2 mmol/l glucose (2G) or with 20 mmol/l glucose (20G) with or without 20 mmol/l KCl. *n* = 9–12, where an *n* of 1 equals one perifusion of 160 islets isolated from 4–6 mice. (**l**, **m**) Glucose-stimulated [Ca^2+^]_cyt_ was measured in isolated mouse islets treated with 1 or 5 ng/ml eNAMPT-WT for 48 h (*n* = 9–16 islets/treatment from three mice). Calcium data are expressed as F/F minimum, where F is fluorescence at any given time point and F minimum is the minimum fluorescence during the recording. For (**h**–**m**): black, 0 ng/ml eNAMPT; blue, 1 ng/ml eNAMPT-WT; grey, 5 ng/ml eNAMPT-WT. (**n**) Static insulin secretion in response to basal (2 mmol/l) or 20 mmol/l glucose was assessed in isolated mouse islets incubated with 1 ng/ml eNAMPT-WT ± 1 ng/ml eNAMPT monomer for 48 h (*n* = 4–19). Black bars, 2 mmol/l glucose; grey bars, 20 mmol/l glucose. Data are expressed as means ± SEM. *p < 0.05, **p < 0.01, ***p < 0.001, as indicated. In (**e**): ***p < 0.001 vs 1 ng/ml eNAMPT-WT; ^†††^*p* < 0.001 vs 0 ng/ml eNAMPT-WT; ^‡^*p* < 0.05, ^‡‡^*p* < 0.01 vs 0 ng/ml eNAMPT monomer. In (**h**) and (**j**): *p < 0.05, **p < 0.01, ***p < 0.001, 0 ng/ml vs 1 ng/ml eNAMPT; ^†^*p* < 0.05, ^††^*p* < 0.01, 1 ng/ml vs 5 ng/ml eNAMPT. Data analysed by two-way ANOVA with Tukey’s post hoc test (**a**–**g**, **n**) or one-way ANOVA with Bonferroni’s post hoc test (**h**–**m**)

eNAMPT-WT induced a bimodal response; 1 ng/ml eNAMPT significantly enhanced static glucose-stimulated insulin secretion (GSIS) in mouse and human islets (24–48 h; Fig. 2a, b, e, f), and enhanced dynamic GSIS (48 h; Fig. 2h, i) and intracellular cytosolic calcium levels ([Ca^2+^]_cyt_) (Fig. 2l, m; AUC, *p* < 0.01) in mouse islets. In contrast, neither 5 ng/ml eNAMPT-WT nor eNAMPT monomer (0.1–5 ng/ml; 48 h) stimulated static or dynamic GSIS or increased intracellular calcium in mouse or human islets, with similar or reduced levels of these variables compared with islets not treated with eNAMPT (Fig. 2a–m). A similar bimodal insulin-secretory response was observed following exposure to commercial eNAMPT (Adipogen, Seoul, South Korea; ESM Fig. 3a-c).

We also assessed effects of a combination of eNAMPT-WT and eNAMPT monomer, which is more representative of normal (patho)physiology. eNAMPT-WT (1 ng/ml) alone induced GSIS as expected; however, when eNAMPT-WT (1 ng/ml) was added in combination with eNAMPT monomer, the insulin stimulatory effects of eNAMPT-WT were lost (Fig. 2n).

Together this demonstrates that low, physiological levels of eNAMPT enhance beta cell function, but as eNAMPT rises to pathophysiological levels (~5 ng/ml), beneficial effects are lost and eNAMPT mediates beta cell dysfunction. The effects of high concentrations of eNAMPT-WT are mimicked by non-dimerising eNAMPT monomer, consistent with the notion that eNAMPT-WT adopts monomeric structure and function at higher concentrations.

### Pathophysiological eNAMPT reduces beta cell number and identity and increases alpha cell number

**Fig. 3:**
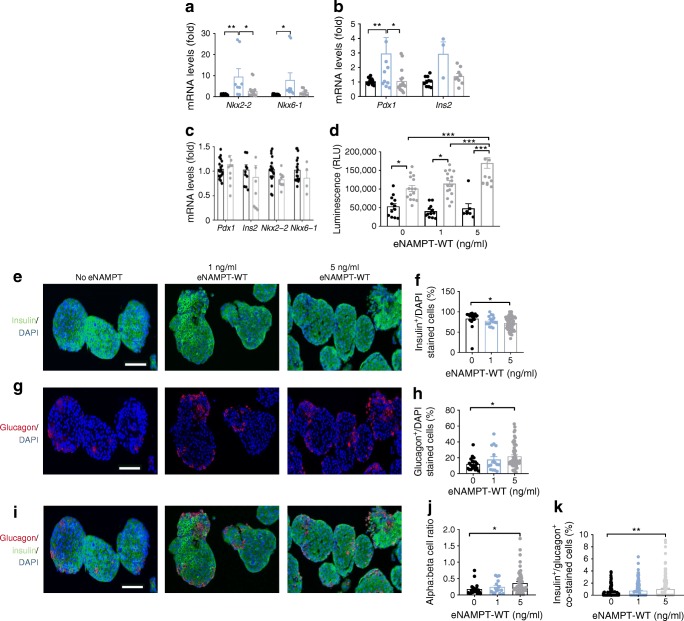
Pathophysiological eNAMPT mediates reductions in beta cell identity and number. (**a**–**c**) Gene expression of *Nkx2-2*, *Nkx6-1*, *Pdx1* and *Ins2* was measured in mouse islets treated with (**a**, **b**) 1 ng/ml eNAMPT-WT (blue bars), 5 ng/ml eNAMPT-WT (grey bars), or (**c**) 1 ng/ml eNAMPT monomer (grey bars) for 48 h. In (**a**–**c**), black bars, untreated. (**d**) Apoptosis (caspase 3/7 activity) was measured in islets treated with eNAMPT-WT with (grey bars) and without (black bars) a cocktail of cytokines (TNF-α, IFNγ and IL-1β; *n* = 7–16, where an *n* of 1 equals one well with six size-matched islets); (**e**–**k**) Mouse islets were treated with 1 or 5 ng/ml eNAMPT-WT for 48 h and assessed by immunofluorescence. (**e**) Double immunofluorescence images of islets stained for insulin (green) and DAPI (blue) and (**f**) bar chart showing per cent of insulin^+^/DAPI cells. (**g**) Double immunofluorescence images of islets stained for glucagon (red) and DAPI (blue) and (**h**) bar chart showing per cent of glucagon^+^/DAPI stained cells. (**i**) Immunofluorescence images of islets stained for insulin (green), glucagon (red) and DAPI (blue) and (**j**) bar chart showing per cent DAPI/glucagon^+^:DAPI/insulin^+^ cells. (**k**) Per cent of insulin^+^/glucagon^+^ bi-hormonal cells. For (**e**–**k**), *n* = 3, where *n* of 1 equals 200–300 islets from 1 mouse. Data are expressed as means ± SEM. *p < 0.05, **p < 0.01, ***p < 0.001, by two-way ANOVA with Tukey’s post hoc test (**a**, **b**, **d**) or one-way ANOVA with Bonferroni’s post hoc test (**c**, **f**, **h**, **j**, **k**)

Type 2 diabetes is characterised by reduced beta cell mass, with loss of beta cell identity and increased apoptosis as possible mechanisms. Consistent with the bimodal insulin-secretory response, mRNA levels of islet *Ins2* (*p* > 0.05) and the essential beta cell identity markers [35] *Pdx1* (*p* < 0.01), *Nkx2-2* (*p* < 0.01) and *Nkx6-1* (*p* < 0.05) were increased in mouse islets by 1 ng/ml eNAMPT-WT but not by 5 ng/ml eNAMPT-WT, nor 1 ng/ml eNAMPT monomer (48 h; Fig. 3a–c), as compared with untreated islets. Treatment with 5 ng/ml eNAMPT-WT also enhanced cytokine-mediated islet apoptosis, (caspase 3/7 activity) (*p* < 0.001 vs no eNAMPT; Fig. 3d), as well as significantly reducing insulin^+^ cells (*p* < 0.05) and increasing glucagon^+^ cells (*p* < 0.05) and non-significantly increasing somatostatin^+^ cells, denoting decreased beta cell and increased alpha and delta cell number, respectively (Fig. 3e–k; ESM Fig. 4).

### eNAMPT impairs beta cell function via proinflammatory mechanisms

**Fig. 4:**
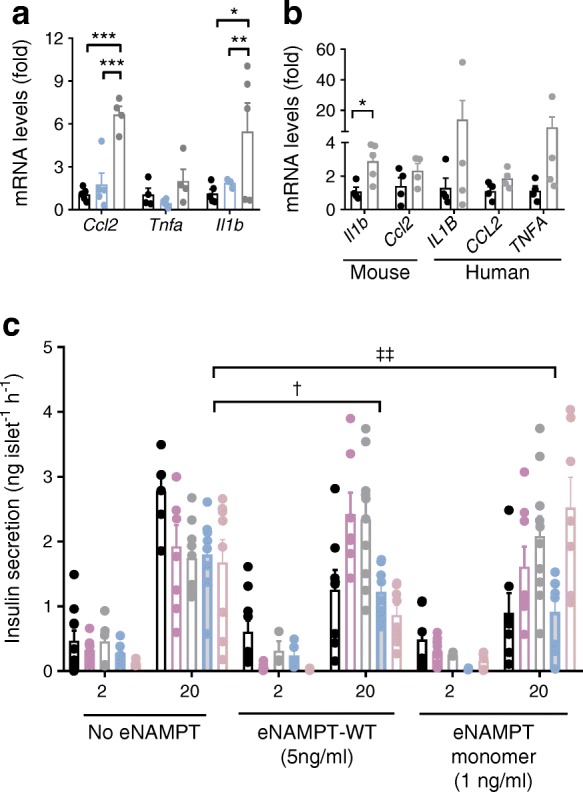
Monomeric eNAMPT impairs GSIS via proinflammatory mechanisms. (**a**) Gene expression of *Ccl2*, *Tnfa* and *Il1b* in mouse islets after treatment with eNAMPT-WT at 1 ng/ml (blue bars) or 5 ng/ml (grey bars) (black bars, untreated); *n* = 4–5. (**b**) Gene expression of *Il1b/IL1B*, *Ccl2/CCL2* and *TNFA* in mouse and human islets after treatment with 1 ng/ml eNAMPT monomer (grey bars) (black bars, untreated); *n* = 4–5. (**c**) Mouse islets were treated with 5 ng/ml eNAMPT-WT or 1 ng/ml eNAMPT monomer in combination with inhibitors of P38-MAPK (SB203580; 1 μmol/l; purple bars), STAT3 (NCS74859; 50 μmol/l; grey bars), JNK (SP600125; 50 μmol/l; blue bars) or NF-κB (BAY 11–7082; 1 μmol/l; brown bars) for 48 h (no inhibitor, black bars), and insulin secretion was measured after static incubation with 2 mmol/l or 20 mmol/l glucose (*n* = 8–10). In (**a**–**c**), an *n* of 1 equals 150–250 islets. Data are expressed as means ± SEM. *p < 0.05, **p < 0.01, ***p < 0.001, as indicated. In (**c**): ^†^*p* < 0.05 for islets treated with JNK inhibitor and 5 ng/ml eNAMPT-WT vs islets treated with JNK inhibitor without eNAMPT; ^‡‡^*p* < 0.01 for islets treated with JNK inhibitor and 1 ng/ml eNAMPT monomer vs islets treated with JNK inhibitor without eNAMPT. Data analysed by two-way ANOVA with Sidak’s post hoc test

Proinflammatory effects of eNAMPT are reported, although, to date, not in type 2 diabetes. eNAMPT-WT at 5 ng/ml (48 h), but not at 1 ng/ml, increased mouse islet *Il1b* (*p* < 0.05) and *Ccl2* (*p* < 0.001) gene expression, as compared with untreated islets (Fig. 4a). Similarly, eNAMPT monomer (1 ng/ml; 48 h) increased *IL1B/Il1b* and *CCL2/Ccl2* gene expression in mouse and human islets (significant [*p* < 0.05] for *Il1b* only) (Fig. 4b). Inflammatory pathway inhibitors demonstrated that inhibition of STAT3 and P38-MAPK, but not of NF-κB or JNK (inhibition of which still resulted in reduced GSIS; *p* > 0.05 for NF-κB and *p* < 0.05 for JNK), blocked the inhibitory effects of eNAMPT on GSIS (Fig. 4c).

Therefore, pathophysiological concentrations of eNAMPT-WT and eNAMPT monomer induce beta cell dysfunction, in part through promotion of STAT3 and P38-mediated islet inflammation.

### Structure-functional changes mediate bimodal effects of eNAMPT-WT

Since the effects of eNAMPT monomer were mimicked by 5 ng/ml, but not 1 ng/ml eNAMPT-WT, we hypothesised that high concentrations of eNAMPT-WT (5 ng/ml) would be associated with reduced NAD-biosynthetic capacity (compared with 1 ng/ml eNAMPT-WT), indicative of reduced eNAMPT dimer and increased eNAMPT monomer levels.

**Fig. 5:**
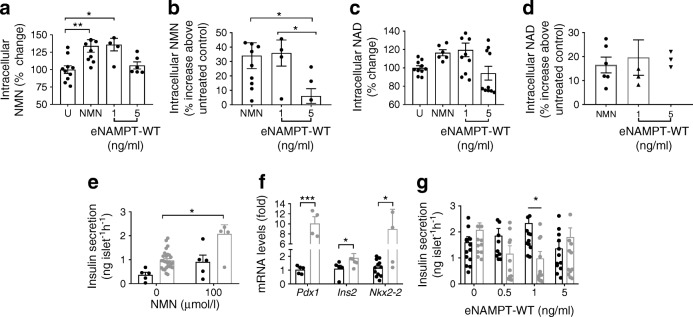
Structure-functional changes mediate the bimodal effects of eNAMPT-WT. (**a**–**d**) MIN6 cells were incubated with either NMN (100 μmol/l) or eNAMPT-WT (1 or 5 ng/ml) for 48 h. (**a**, **b**) intracellular NMN and (**c**, **d**) intracellular NAD. In (**a**–**d**), *n* = 5–10, where *n* of 1 equals 1 well of a 12-well plate. (**e**, **f**) Mouse islets were treated with NMN (100 μmol/l), and (**e**) static insulin secretion in response to 2 mmol/l glucose (black bars) or 20 mmol/l glucose (grey bars) was analysed (*n* = 5–28, where an *n* of 1 equals five islets per incubation tube, repeated 8–10 times) and (**f**) gene expression of *Pdx1*, *Ins2* and *Nkx2-2* was measured by quantitative RT-PCR (*n* = 5–12, where an *n* of 1 equals RNA extracted from 150–200 islets). (**g**) Mouse islets were treated for 48 h with eNAMPT-WT (0.5, 1 or 5 ng/ml) in combination with the CD73 inhibitor AMP-CP (1 μmol/l) and static insulin secretion was measured in response to 20 mmol/l glucose (*n* = 17, where an *n* of 1 equals five islets per incubation tube, repeated 8–10 times). In (**f**) and (**g**): black bars, 0 μmol/l NMN; grey bars, 100 μmol/l NMN. Data are expressed as means ± SEM, *p < 0.05, **p < 0.01, ***p < 0.001 vs untreated cells (in (**b**) and (**d**), untreated cells are at 0%) or as indicated, by one-way ANOVA with Bonferroni’s post hoc test (**a**–**d**) or two-way ANOVA with Sidak’s post hoc test (**e**–**g**). U, untreated

In support of this, treatment of MIN6 cells with 1 ng/ml eNAMPT-WT (48 h) increased cellular NMN (*p* < 0.05) and NAD (*p* > 0.05) vs basal levels, by 30% and 20%, respectively. These changes were mimicked by NMN treatment (100 μmol/l for 48 h; Fig. 5a–d). NMN also mimicked the functional effects of 1 ng/ml eNAMPT-WT, significantly increasing levels of GSIS and *Ins2*, *Nkx2-2* and *Pdx1* mRNA (Fig. 5e, f). In contrast, and similar to the effects of eNAMPT monomer, 5 ng/ml eNAMPT-WT did not increase cellular NMN or NAD (Fig. 5a–d and ESM Fig. 2d-e). Furthermore, AMP-CP-mediated inhibition of CD73 (a protein involved in cellular NMN uptake) [36–40] inhibited the insulin-secretory effects of 1 ng/ml eNAMPT-WT (*p* < 0.05) but had no effects on the inhibitory actions of 5 ng/ml eNAMPT-WT on GSIS (Fig. 5g). Thus, 1 ng/ml eNAMPT induces NAD biosynthesis and requires islet NMN uptake to exert its functional effects. In contrast, the effects of 5 ng/ml eNAMPT were unaffected by inhibition of NMN uptake, suggesting an NAD-independent pathway for these effects.

These data provide evidence that the bimodal effects of eNAMPT-WT are related to loss of NAD-biosynthetic capacity of eNAMPT at higher concentrations.

## Discussion

This study demonstrates that eNAMPT exerts bimodal, concentration- and structure-functional-dependent effects on pancreatic beta cell functional mass, and provides clarification of previous contradictory eNAMPT studies.

Low, physiological levels of eNAMPT (1 ng/ml) enhance beta cell health through NAD-dependent mechanisms. Since islets are continually exposed to low eNAMPT concentrations in healthy non-diabetic individuals, this indicates a role for low eNAMPT concentrations in maintaining beta cell health. Beta cell identity genes were increased at 1 ng/ml eNAMPT, highlighting the potential importance of physiological levels of eNAMPT in maintaining beta cells in the fully differentiated state. This is consistent with studies showing beta cell de-differentiation following 3-day culture in the absence of circulating factors, including eNAMPT/NMN [41]. The effects of 1 ng/ml eNAMPT are mediated via NAD biosynthesis, suggesting eNAMPT exists in the dimeric form at lower concentrations. This is consistent with our observations in non-diabetic human and mouse serum samples, which show eNAMPT dimer as the predominant structural form of eNAMPT at low physiological concentrations [12]. Whether eNAMPT can exert NAD biosynthesis within the extracellular space remains controversial. Whilst RPMI media contains sufficient nicotinamide to support eNAMPT enzymatic activity, we were unable to detect eNAMPT within spent islet media after eNAMPT incubation, suggesting that at least some eNAMPT dimer is taken up into the islet/beta cell, after which NMN synthesis occurs intracellularly.

Mechanistically, eNAMPT-mediated NAD synthesis may improve beta cell health through activation of NAD-dependent sirtuins or via actions of NAD metabolites cyclic ADP-ribose (cADPR) and nicotinic acid adenine dinucleotide phosphate (NAADP), which reportedly induce intracellular calcium mobilisation [13, 14, 16, 19].

Conversely, as eNAMPT concentrations rise to pathophysiological levels, as in type 2 diabetes, eNAMPT induces islet inflammation, and initiates beta cell dysfunction, beta cell death and reduced beta cell mass and identity. The mechanisms driving reduced beta cell number and identity are unclear. We have demonstrated that high concentrations of eNAMPT enhance beta cell apoptosis, however beta to alpha cell trans-differentiation or beta cell de-differentiation may also play a role, and further studies are required to identify the precise mechanisms.

eNAMPT exerts beta cell dysfunction through NAD-independent mechanisms, suggesting a switch toward monomeric eNAMPT at higher concentrations. This is consistent with observations that the eNAMPT monomer was present at 1–2 ng/ml in serum from individuals with type 2 diabetes, but almost absent in serum from individuals without diabetes. It is also consistent with our observations that increasing serum eNAMPT did not correlate with increasing serum NMN.

Whilst elevated eNAMPT strongly correlated with increased HbA_1c_, we did not observe notable correlations between eNAMPT and BMI, and only a weak correlation between eNAMPT and serum insulin. We hypothesise that, as glucose rises during glucose intolerance, secretion of eNAMPT dimer increases, which promotes islet compensation. However, as eNAMPT levels continue to rise in response to elevated glucose, eNAMPT dimer begins to break apart into constituent eNAMPT monomers, which then drive beta cell failure. Thus, increases in eNAMPT strongly correlate with increases in blood glucose and HbA_1c_ and do not necessarily precede hyperglycaemia. Precisely why eNAMPT preferentially exists as a monomer at high levels remains unclear but may relate to ligand-induced dimerisation, whereby eNAMPT is stabilised and maintained in dimer form by the presence of an endogenous ligand. We hypothesise that insufficient concentrations of the putative ligand at higher eNAMPT concentrations lead to break up of the dimer into constitutive monomers.

Mechanistically, eNAMPT promoted islet inflammation and beta cell failure through P38-MAPK and STAT3 pathways. Similar eNAMPT effects are reported in macrophages, monocytes and islets of mice fed a high-fat diet (HFD) [7, 9, 12, 42]. Upstream, these effects may be receptor-mediated, with eNAMPT reported to function via Toll-like receptor 4 (TLR4) [43], C-C chemokine receptor type 5 (CCR5) [44], IGF and insulin receptor [8] signalling, although these remain unconfirmed. Separately, the deleterious effects of eNAMPT monomer may also reflect decreased eNAMPT dimer-mediated NAD synthesis, occurring independently from direct effects of eNAMPT monomer.

Together, our results characterise structure-functional relationships as being of crucial importance to the effects of eNAMPT on beta cell health. Moreover, we demonstrate a novel mechanism of beta cell dysfunction in type 2 diabetes. Strategies to block the actions of the eNAMPT monomer by promoting dimerisation or stabilising eNAMPT in dimer form could represent promising therapeutic approaches for the treatment of diabetes.

## Electronic supplementary material

ESM (PDF 776 kb)

## Data Availability

Data are available on request from the authors.

## Abbreviations

AMP-CP: Adenosine 5′-(α,β-methylene)diphosphate
eNAMPT: Extracellular nicotinamide phosphoribosyltransferase
eNAMPT-WT: Recombinant wild-type extracellular nicotinamide phosphoribosyltransferase
FPG: Fasting plasma glucose
GSIS: Glucose-stimulated insulin secretion
IFG: Impaired fasting glucose
iNAMPT: Intracellular nicotinamide phosphoribosyltransferase
JNK: c-Jun N-terminal kinase
MAPK: Mitogen-activated protein kinase
NAMPT: Nicotinamide phosphoribosyltransferase
NMN: Nicotinamide mononucleotide
STAT3: Signal transducer and activator of transcription 3
WT: Wild-type
