# Supplementary material for: Structure-functional changes in eNAMPT at high concentrations mediate mouse and human beta cell dysfunction in type 2 diabetes
Source: Diabetologia. 2019 Nov 15;63(2):313–23. doi: 10.1007/s00125-019-05029-y (PMC6946736; doi:10.1007/s00125-019-05029-y)
Supplement: Supplementary file 1 — (PDF 776 kb) [file 125_2019_5029_MOESM1_ESM.pdf]

ESM Methods

Human samples

Human serum samples were obtained from obese non-diabetic individuals (BMI: 46.4 +/- 1.8 kg/m^2; FPG: <5.6 mmol/L; Age: 53.0 +/- 2.0 years; n=13) and obese individuals with IFG (BMI: 37.5 +/- 1.9 kg/m^2; FPG 5.6 – 6.9 mmol/L; Age: 54.9 +/- 3.3 years; n=15) and type 2 diabetes (BMI: 36.3 +/- 1.6 kg/m^2; FPG ≥7.0 mmol/L; Age: 41.5 +/- 3.5 years; n=27), as part of the Body Fat, Surgery and Hormone study (BodyFatS&H) conducted at the University of Auckland, New Zealand (NTX/08/10/103; Northern Regional Ethics Committee, Auckland, New Zealand) and as part of the VaSera trial [1], conducted at St Thomas' Hospital, London (ISRCTN25003627; NRES Committee-London Central, 21/12/2012, ref: 12/LO/1850). VaSera trial participants consisted of individuals with type 2 diabetes or those at risk of developing type 2 diabetes (FPG: 5.6 – 6.9 mmol/L; BMI >30 kg/m^2).

Native Mass Spectrometry

The protein was expressed and isolated as previously described and, on the day of analysis, the buffer was exchanged into 100 mM ammonium acetate (Fisher Scientific, Loughborough, UK) pH 6.9 using micro Bio-Spin Chromatography columns (Micro Bio-Spin 6 Columns, Bio-Rad, Watford, UK) following the instructions specified by the manufacturer. The procedure was repeated twice and diluted to give a final concentration of WT NAMPT (5 μmol/l) or S200D NAMPT (5 μmol/l). Native MS data was acquired on the Synapt G2S HDMS (Waters, Manchester, UK). NanoESI capillaries were prepared in-house from thin-walled borosilicate capillaries (inner diameter 0.9 mm, outer diameter 1.2 mm, World Precision Instruments, Stevenage, UK) using a Flaming/Brown P-1000 micropipette puller (Sutter Instrument Company, Novato, CA, USA). A positive voltage was applied to the solution via a platinum wire (Goodfellow Cambridge Ldt, Huntington, UK) inserted into the capillary. Gentle source conditions were applied to preserve the native-like structure: capillary voltage 1.2-1.5 kV, sampling cone 50-200 V, source temperature 70 °C. Trap collision energy was 4 V, transfer collsion energy was set to 0 V. Nitrogen was the carrier gas. External calibration of the spectra was achieved using solutions of cesium iodide (2 mg/mL in 50:50 water:isopropanol). Data were acquired and processed with MassLynx software (Waters, Manchester, UK).

eNAMPT protein generation

Construction of DNA plasmids: DNA for full length mouse NAMPT was synthesised as a double stranded DNA gBlock (Integrated DNA Technologies) and cloned into pET151 according to manufacturer's instructions (ThermoFisher). This vector adds an N-terminal 6xHis and V5 tag, and a TEV cleavage site, giving an extra 33 amino acids on the N-terminus of the protein. Mutagenesis was carried out to change residues S^{199} and S^{200} to D^{199} and D^{200} using the overlapping primers GGTTACAGAGGAGTCGACGATCAAGAGACTGCTGGC and GCCAGCAGTCTCTTGATCGTCGACTCCTCTGTAACC, and the Quikchange lightning kit (Agilent Technologies) according to the manufacturer's instructions. The constructs and mutations were verified by sequencing (Eurofins Genomics).

Expression of NAMPT wild type and eNAMPT-monomer (SS199/200DD) mutation: For expression, the constructs were transformed into BL21 Star (DE3) *E. coli* (ThermoFisher). Colonies were used to inoculate a starter culture in Luria Broth containing 100ug/mL ampicillin, and left shaking at 37°C for 5 hours. This was used to inoculate 200mL ZYP-5052 autoinduction media (Studier, 2005) and the culture grown at 18°C shaking for 65 hours. The bacteria were harvested by centrifugation at 4000g and frozen at -80°C.

Purification of eNAMPT-WT and eNAMPT-WT (SSDD) mutation: Pellets were thawed and EDTA Complete protease inhibitor tablets added (Roche, Welwyn Garden City, UK) then the *E. coli* were lysed using BugBuster (Merck Millipore, Burlington, MA, USA) according to the manufacturer's instructions. The NAMPT was found in the soluble fraction and was purified by passing over a 1mL Histrap ff crude column (GE Healthcare, Chicago, IL, USA) using a Biorad NGC system. The column was washed with 30 column volumes of 10mM sodium phosphate, 500 mmol/l NaCl, 20 mmol/l imidazole pH 7.4 and the bound protein eluted with 10 mmol/l sodium phosphate, 500 mmol/l NaCl, 500 mmol/l imidazole pH 7.4. Fractions containing protein were then pooled and concentrated (Amicon Ultra 15, Merck Millipore), then further purified using size exclusion chromatography. Using a Gilson HPLC system, samples were run on a Superdex 200 increase 10/300 GL column in Phosphate buffered saline pH 7.4 (OXOID). Fractions corresponding to monomer and dimer were pooled individually. Final yield of protein was approximately 40mg/L for the WT, 8mg/L for monomer SSDD fraction and 5mg/L dimer SSDD fraction.

Pancreatic islet isolation

Mouse CD1 Islets were isolated as described previously [2]. Pancreata were inflated with 1mg/mL collagenase solution (Sigma-Aldrich, Poole, U.K.) followed by density gradient separation (Histopaque-1077; Sigma-Aldrich). Human islets were isolated from heart-beating non-diabetic donors, with appropriate ethical approval, at the King's College Hospital Human Islet Isolation Unit [2]. Isolated islets were incubated overnight (37°C, 5% CO_2) prior to treatments. See human islet checklist table for additional details of human islet preparations.

Static and dynamic glucose-stimulated insulin secretion

For static insulin secretion, mouse CD1 or human islets were pre-incubated in a physiological salt solution [3] containing 2mM glucose. Groups of 3-5 size-matched islets were then further incubated at 37°C for 1 h in salt solution and 2 or 20 mmol/l glucose. Dynamic insulin secretion was measured using a temperature-controlled perifusion system. Mouse CD1 islets were transferred into chambers containing 1 µm pore-size nylon filters (40 islets/chamber) and perifused with a physiological salt solution [3] (37°C, 0.5mL/min) containing 2 or 20 mmol/l glucose or 20 mmol/l glucose and 20 mmol/l KCl. Samples were collected at 2-minute intervals throughout the experiment. Secreted insulin was measured using an in-house I^{125} radioimmunoassay [3].

Quantitative RT-PCR

Total mouse CD1 or human islet RNA was extracted using Trizol reagent (Invitrogen, Paisley, UK). Reverse transcription was performed using the High-Capacity cDNA reverse transcription kit (Applied Biosystems, Warrington, UK). Real-time qPCR was carried out with a LightCycler480, using Sybr Green PCR master mix (Qiagen, Hilden, Germany). Gene expression was measured by ΔΔCt methodology, normalised against GAPDH (Quantitech, UK). For primer details (all Eurogentec, Southampton, UK) see ESM Table 1.

NMN measurements

NMN was measured in MIN6 cells and human serum using a fluorometric assay based on previous described methodology [4,5]. MIN6 or serum samples (30 µL) were extracted with 100 µL perchloric acid (1 mol/L) and then neutralized by addition of 330 µL K_2CO_3 (3 mol/L) followed by incubation at

4°C for 10 min, and centrifugation at 12000 x g for 15 min at 4°C, with the supernatant retained. Serum and cellular samples (50 μL) and standard solutions of NMN (50 μl; 25 – 200 μmol/l) were subsequently derivatised by addition of 20 μl KOH (1 mol/L) and 20 μl acetophenone (Sigma, Poole, UK) followed by incubation at 4°C for 15 min. Formic acid (90 μL) was then added and the solution incubated for 10 min at 37°C, producing a highly fluorescent compound. Samples or standards (150 μL) were then added into a 96-well plate fluorescence was detected on a SpectraMax i3x plate reader with excitation and emission wavelength of 382 and 445 nm, respectively.

eNAMPT Immunoblotting

Serum non-reducing immunoblotting was conducted as previously described, with samples run on SDS-PGE gels without reducing agent. Blocking was carried out using 5% (wt/vol) milk protein. A rabbit anti-mouse polyclonal primary antibody against NAMPT (Catalogue number, D7V5J; Cell Signalling Technologies, MA, USA) was used to detect NAMPT (1:1000 dilution in Tris-buffered saline buffer containing 5% (vol/vol) Tween-20). NAMPT-bound primary antibody was detected using anti-rabbit IgG HRP-linked secondary antibody (Catalogue number, 7074S; Cell Signalling Technologies) at 1:3000 dilution in Tris-buffered saline buffer containing 5% (vol/vol) Tween-20). Immunoblots were conducted under non-reducing conditions, where no reducing agent was added to the protein samples prior to SDS-PAGE. This allows detection of both monomeric and dimeric eNAMPT in biological samples. Densitometry of western blot bands was calculated using Image J Software and densitometry data was used to calculate percentage ratio of eNAMPT monomer:dimer in ND and T2D serum. Concentrations of monomer and dimer in T2D serum were calculated by taking the total eNAMPT concentration present in the sample (as calculated by ELISA) and using the percentages derived from densitometry to calculate final serum concentrations of monomer and dimer. Western blots are representative of 3 separate blots. sTo ensure equivalent loading of each gel lane, serum protein was equalized to 10 μg and an equal volume (30 μl) was added to each well.

References

1. Mills CE, Govoni V, Faconti L, Casagrande ML, Morant SV, Webb AJ, et al. Reducing Arterial Stiffness Independently of Blood Pressure: The VaSera Trial. Journal of the American College of Cardiology. 2017;70(13):1683-4.

2. Huang GC, Zhao M, Jones P, Persaud S, Ramracheya R, Lobner K, et al. The development of new density gradient media for purifying human islets and islet-quality assessments. Transplantation. 2004;77(1):143-5.

3. Jones PM, Salmon DM, Howell SL. Protein phosphorylation in electrically permeabilized islets of Langerhans. Effects of Ca2+, cyclic AMP, a phorbol ester and noradrenaline. The Biochemical journal. 1988;254(2):397-403.

4. Formentini L, Moroni F, Chiarugi A. Detection and pharmacological modulation of nicotinamide mononucleotide (NMN) in vitro and in vivo. Biochemical pharmacology. 2009;77(10):1612-20.

5. Zhang RY, Qin Y, Lv XQ, Wang P, Xu TY, Zhang L, et al. A fluorometric assay for high-throughput screening targeting nicotinamide phosphoribosyltransferase. Analytical biochemistry. 2011;412(1):18-25.

ESM Table 1

**Human primers**		
Gene	Forward primer (5'-3')	Reverse primer (3'-5')
IL1B	GGCTGCTCTGGGATTCTCTT	CCATCATTTCACTGGCGAGC
CCL2	CACCTGGACAAGCAAACCCA	GTGTCTGGGGAAAGCTAGGG
TNFA	GCCCATGTTGTAGCAAACCC	TATCTCTCAGCTCCACGCCA
**Mouse primers**		
Gene	Forward primer (5'-3')	Reverse primer (3'-5')
Gapdh	AGGGCTGCTTTTAACTCTGGT	CCCCACTTGATTTTGGAGGGA
Pdx1	GAACCCGAGGAAAACAAGAGG	GTTCAACATCACTGCCAGCTC
Ins2	CCGTGGTGAAGTGGAGGA	CAGTTGGTAGAGGGAGCAGAT
Nkx2.2	CCTTTCTACGACAGCAGCGA	CCGTGCAGCGAGTATTGCAG
Nkx6.1	CAAGGCTGCACATCGTGTTT	GAACAGGCTAGGTGGGTCTG
Il1b	GGGCTGCTTCCAAACCTTTG	TGATACTGCCTGCCTGAAGCTC
Ccl2	GGCTGGAGAGCTACAAGAGG	GGTCAGCACAGACCCTCTCTG
Tnfa	CGGAGTCCGGGCAGGT	GCTGGGTAGAGAATGGATGAACA

ESM Table 1. Table of mouse and human qPCR primers

ESM Figures

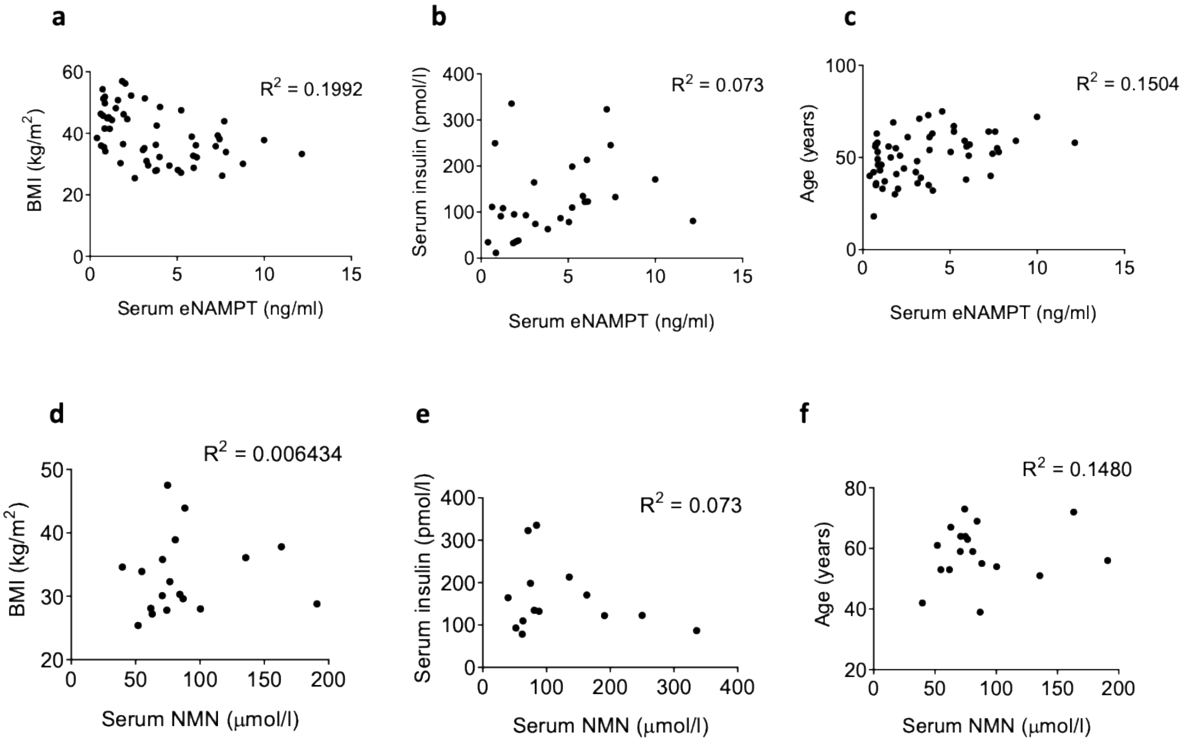

ESM Figure 1: Serum eNAMPT and NMN levels are not correlated with BMI or age. Serum was collected from non-diabetic, IFG and type 2 diabetic individuals, and eNAMPT and NMN were measured by ELISA and fluorometric assay, respectively**. (a)** Serum eNAMPT vs BMI (data generated from BodyFatS&H and VaSera trial samples); **(b)** Serum eNAMPT vs serum insulin; **(c)** Serum eNAMPT vs. age (data generated from BodyFatS&H and VaSera samples). **(d)** Serum NMN vs BMI (data generated from BodyFatS&H and VaSera trial samples); **(e)** Serum NMN vs serum insulin; **(f)** Serum NMN vs age (NMN data generated from VaSera samples only). N is equal to 1 individual. N values differ for NMN measurements due to limited sample availability for some samples. Statistics were calculated using Pearson correlation.

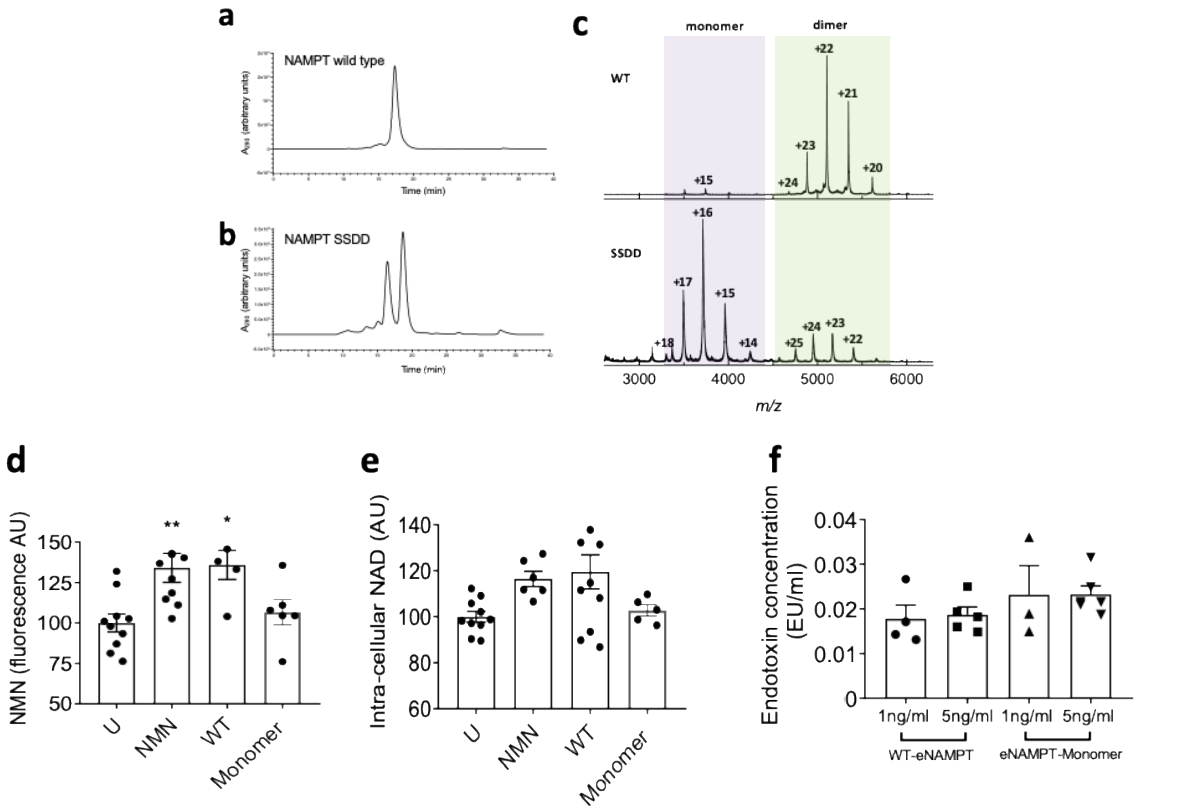

ESM Figure 2: Structural and functional characterisation of eNAMPT-monomer and eNAMPT-WT. **(a-b)** Size exclusion chromatography data showing **(a)** eNAMPT-WT and **(b)** eNAMPT-monomer (SSDD); **(c)** Native mass spectra of 6.5 μM eNAMPT-WT and 5 μM eNAMPT-monomer (SSDD) in 100 mM ammonium acetate showing differences in the monomer to dimer distribution. **(D – E)** MIN6 cells were incubated with either NMN (100 μM), WT-eNAMPT (1 ng/mL) or eNAMPT-monomer (1 ng/mL) for 48 hours; (**d**) intra-cellular NMN, **(e)** intra-cellular NAD (*n*=5 – 10). **(f)** Endotoxin concentrations in preparations of WT-eNAMPT and eNAMPT-monomer. Data are expressed as means ± SEM. *P<0.05, **P<0.01 by 1-way ANOVA followed by Tukey's post-test

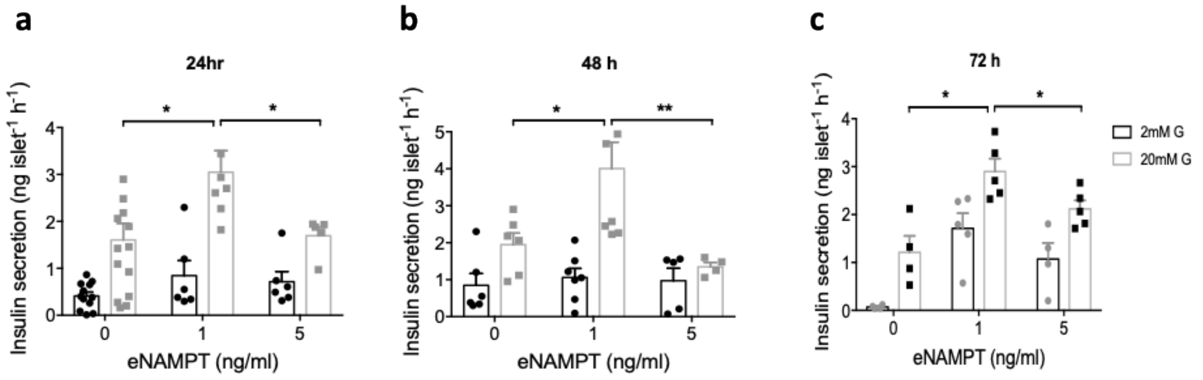

ESM Figure 3: Commercially obtained eNAMPT exerts a bi-phasic effect on glucose-stimulated insulin secretion Static insulin secretion in response to basal (2mM) or 20mM glucose was assessed in isolated mouse islets incubated with 1 – 5 ng/mL commercially obtained eNAMPT (Adipogen, Seoul, South Korea) for **(a)** 24 hours *(n*=12), **(b)** 48 hours *(n*=6) and **(c)** 72 hours *(n*=5). Data are expressed as mean ± SEM, *P<0.05, **P<0.01, by 2-way ANOVA followed by Sidak's post-test.

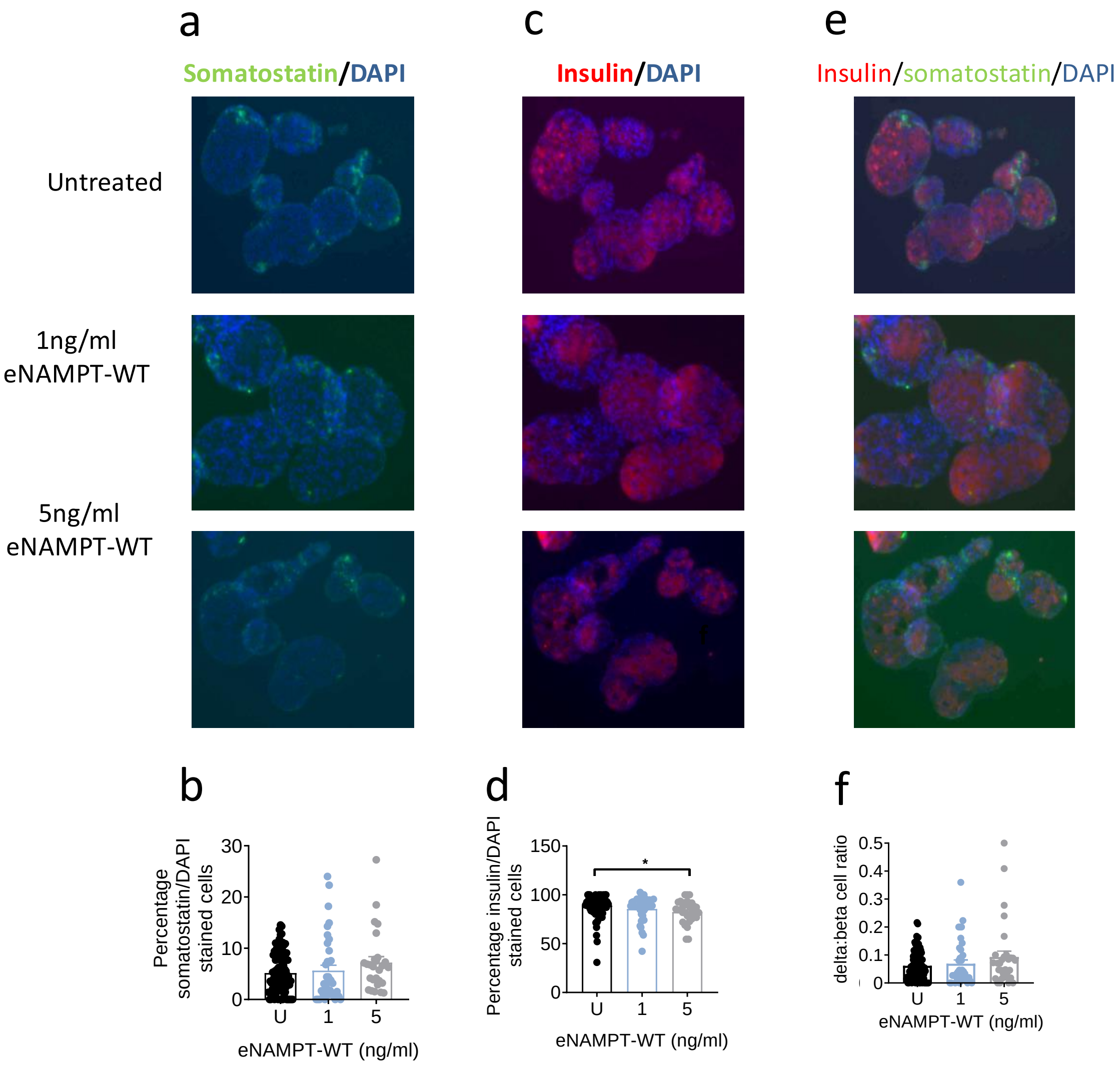

ESM Figure 4: Effects of eNAMPT-WT on islet delta-cell number. Mouse islets were treated with 1 – 5 ng/mL eNAMPT-WT for 48 h **(a)** Double immunofluorescence images of islets stained for somatostatin (green) and DAPI (blue) **(b)** % DAPI/SST positive stained cells *(n*=3); **(c)** Double immunofluorescence images of islets stained for insulin (red) and DAPI (blue) **(d)** % DAPI/INS positive stained cells (*n*=3); (**e**) Immunofluorescence images of islets stained for SST (green), INS (red) and DAPI (blue) **(f)** % DAPI/SST:% DAPI/INS stained cells (*n*=3); Data are expressed as means ± SEM, *P<0.05, by 1-way ANOVA with Tukey's post-test.

Checklist for reporting human islet preparations used in research

Adapted from Hart NJ, Powers AC (2018) Progress, challenges, and suggestions for using human islets

to understand islet biology and human diabetes. Diabetologia https://doi.org/10.1007/s00125-018-4772-2

Islet preparation	1	2	3	4	5
**MANDATORY INFORMATION**					
Unique identifier	Unknown	Unknown	Unknown	Unknown	Unknown
Donor age (years)	50	49	26	49	59
Donor sex (M/F)	Male	Male	Male	Female	Female
Donor BMI (kg/m^2)	Unknown	Unknown	Unknown	Unknown	24
Donor HbA_{1c} or other measure of blood glucose control	Unknown	Unknown	Unknown	Unknown	Unknown
Origin/source of islets[b]	King's College Hospital islet transplantation unit	King's College Hospital islet transplantation unit	King's College Hospital islet transplantation unit	King's College Hospital islet transplantation unit	King's College Hospital islet transplantation unit
Islet isolation centre	King's College Hospital islet transplantation unit	King's College Hospital islet transplantation unit	King's College Hospital islet transplantation unit	King's College Hospital islet transplantation unit	King's College Hospital islet transplantation unit
Donor history of diabetes? Please select yes/no from drop down list	No	No	No	No	No
Diabetes duration (years)	N/A	N/A	N/A	N/A	N/A
Glucose-lowering therapy at time of death[c]	N/A	N/A	N/A	N/A	N/A
**RECOMMENDED INFORMATION**					
Donor cause of death	Unknown	Donation after brain death	Donation after circulatory death	Unknown	Unknown
Warm ischaemia time (h)	Unknown	Unknown	Unknown	Unknown	Unknown
Cold ischaemia time (h)	Unknown	Unknown	Unknown	Unknown	Unknown
Estimated purity (%)	75-80	55	80	70	Unknown
Estimated viability (%)	85	80	80	80	Unknown
Total culture time (h)[d]	72 hours	72 hours	72 hours	120 hours	72 hours
Glucose-stimulated insulin secretion or other functional measurement[e]	Glucose-stimulated Insulin secretion and RNA extraction for qPCR	RNA extraction for qPCR	Glucose-stimulated Insulin secretion	Glucose-stimulated Insulin secretion and RNA extraction for qPCR	Glucose-stimulated Insulin secretion
Handpicked to purity? Please select yes/no from drop down list	Yes	Yes	Yes	Yes	Yes

Additional notes					

[a]If you have used more than eight islet preparations, please complete additional forms as necessary

[b]For example, IIDP, ECIT, Alberta IsletCore

[c]Please specify the therapy/therapies

[d]Time of islet culture at the isolation centre, during shipment and at the receiving laboratory

[e]Please specify the test and the results
